# Machine Learning-Based Prediction of Muscle Injury Risk in Professional Football: A Four-Year Longitudinal Study

**DOI:** 10.3390/jcm14228039

**Published:** 2025-11-13

**Authors:** Francisco Martins, Hugo Sarmento, Élvio Rúbio Gouveia, Paulo Saveca, Krzysztof Przednowek

**Affiliations:** 1 University of Coimbra, CIPER, FCDEFUC, 3004-504 Coimbra, Portugal; joao.martins@staff.uma.pt (F.M.); hg.sarmento@gmail.com (H.S.); 2 Department of Physical Education and Sport, University of Madeira, 9020-105 Funchal, Portugal; 3 LARSYS, Interactive Technologies Institute, 9020-105 Funchal, Portugal; 4 CIPER, Faculty of Human Kinetics, University of Lisbon, Cruz-Quebrada-Dafundo, 1495-751 Lisbon, Portugal; 5 Swiss Center of Expertise in Life Course Research LIVES, 1227 Carouge, Switzerland; 6 Superior School of Sports Sciences, Eduardo Mondlane University, 3453 Maputo, Mozambique; ptiberio_saveca@yahoo.com.br; 7 Mozambique Secondary Institute of Sport and Physical Education, 1071 Maputo, Mozambique; 8 Faculty of Physical Culture Sciences, Medical College, University of Rzeszów, 35-959 Rzeszów, Poland; krzprz@ur.edu.pl

**Keywords:** soccer, injury prediction, classification methods, GPS, RPE

## Abstract

**Background:** Professional football requires more attention in planning work regimens that balance players’ sports performance optimization and reduce their injury probability. Machine learning applied to sports science has focused on predicting these events and identifying their risk factors. Our study aims to (i) analyze the differences between injury incidence during training and matches and (ii) build and classify different predictive models of risk based on players’ internal and external loads across four sports seasons. **Methods:** This investigation involved 96 male football players (26.2 ± 4.2 years; 181.1 ± 6.1 cm; 74.5 ± 7.1 kg) representing a single professional football club across four analyzed seasons. The research was designed according to three methodological sets of assessments: (i) average season performance, (ii) two weeks’ performance before the event, and (iii) four weeks’ performance before the event. We applied machine learning classification methods to build and classify different predictive injury risk models for each dataset. The dependent variable is categorical, representing the occurrence of a time-loss muscle injury (N = 97). The independent variables include players’ information and external (GPS-derived) and internal (RPE) workload variables. **Results:** The Kstar classifier with the four-week window dataset achieved the best predictive performance, presenting an Area Under the Precision–Recall Curve (AUC-PR) of 83% and a balanced accuracy of 72%. **Conclusions:** In practical terms, this methodology provides technical staff with more reliable data to inform modifications to playing and training regimens. Future research should focus on understanding the technical staff’s qualitative vision of predictive models’ in-field applicability.

## 1. Introduction

Football is a physically demanding sport characterized by high-intensity movements that occur intermittently throughout its practice [1,2]. Sprint, agility, power, and strength are specific capacities closely related to success in playing football at an elite level [3,4]. Finding conditioning and working regimens that enhance these actions has become vital for optimizing sports performance and reducing the risk of injury [5,6,7].

Muscle injuries are the most frequent type of injury in professional football, accounting for 20–37% of all injuries that result in player unavailability [8,9]. Additionally, the frequency of injuries impacts players’ professional paths and clubs’ overall performance [10,11]. There is still much to be understood about the causes of muscle injuries in football players [12]. Some investigations involving professional football players have been conducted, providing precise data on muscle injuries necessary for an accurate analysis [13]. However, interpreting such data may not offer coaches and their technical staff sufficient information to determine how to set up training sessions, especially regarding intensity and volume workloads [14].

One of football’s most popular technological advancements is the Global Positioning System (GPS) [15]. This tracking system enables us to measure players’ external loads, which characterize the physical demands imposed on them during their practices and matches. It includes variables such as total distance (TD), high-speed running (HSR), accelerations (ACC), decelerations (DEC), and player load (PL) [16,17,18]. In contrast, internal load refers to the individual’s physiological and psychological responses to these external demands, which are typically assessed through heart rate, hormonal markers, and, in the football context, the Rate of Perceived Exertion (RPE) [19,20,21]. RPE has been commonly applied as a practical, low-cost, and scientifically validated method to quantify internal training load in football and is recognized as a reliable method for monitoring athletes’ physiological responses to exertion [20]. Session-RPE reports significant correlations with objective methods based on heart rate and other physiological indicators in professional players and young athletes [22]. The combined use of GPS (for external load) and RPE (for internal load) enables a comprehensive evaluation of both the imposed workload and the athlete’s response, facilitating a more nuanced understanding of training and match demands and supporting more effective injury prevention strategies [23].

Traditionally, most sports injury research has focused on assessing single variables, such as total distance, the total number of sprints, or the acute-to-chronic workload ratio, to predict the likelihood of an injury occurring [24,25]. However, this univariate approach finds it more challenging to capture the multifactorial nature of injury risk, as it does not account for the complex interactions between workload and physiological and contextual factors [26,27,28]. More recent research has adopted multivariate and machine learning approaches, integrating multiple variables including external load (GPS-derived metrics), internal load (RPE, heart rate), and others, to improve injury prediction accuracy [26,28,29,30]. These multivariate models have demonstrated better predictive performance compared to single-variable models, supporting the need for a comprehensive approach to injury risk assessment [27,28,29].

Machine learning (ML) provides advanced statistical techniques for analyzing nonlinear relationships between multiple variables. It is particularly valuable for injury prediction in football, where risk factors are multifaceted and interact dynamically [27,31,32]. A key challenge in this context is the class imbalance of injury datasets: injuries are rare events compared to the much larger number of non-injury observations, which can cause traditional models to be biased toward predicting the majority (non-injury) class and miss actual injury cases [33,34]. To address this, ML approaches employ strategies such as oversampling, undersampling, and cost-sensitive learning to balance the dataset, ensuring that the model learns to effectively identify injury and non-injury events [31,33]. The performance of ML models is evaluated using metrics such as sensitivity, specificity, and the area under the curve (AUC), which are particularly important in imbalanced datasets [27,31]. This way, we can ensure that proper handling of class imbalance improves the reliability of injury prediction and enhances the practical utility of these models for coaches and medical staff in real-world football settings [32,33].

Therefore, our longitudinal study aims are (i) to analyze the differences between injury incidence during training sessions and official matches and (ii) to build and classify different predictive models of injury risk based on players’ internal and external loads across four sports seasons. This approach is based on the premise that combining external load (the imposed physical demand) and internal load (the physiological response to that demand) provides a more holistic measure of the accumulated stress, which should outperform single-variable predictors in capturing the complexity of injury risk. Given the scarcity of research in this field and the impact that sports injuries have on athletes’ availability and performance, the novelty of our study lies in utilizing a unique, longitudinal four-season dataset, which provides a high-resolution, context-specific analysis of the predictive power of workload variables for injury forecasting. We believe that the period of this investigation in professional football, combined with the application of machine learning techniques to interpret the dataset, can bring greater validity to the sports science literature.

## 2. Materials and Methods

### 2.1. Participants

This investigation had the participation of 96 male football players (26.2 ± 4.2 years; 181.1 ± 6.1 cm; 74.5 ± 7.1 kg) who represented a single professional football club during the timespan of the study and who had continuous evaluations in terms of the external and internal load so that they could be included in the final sample. Of those, 31 were defenders (32.3%), 30 midfielders (31.3%), and 35 forwards (36.4%). Additionally, 21 individuals preferred the left lower limb as the kicking limb (21.9%), while 75 preferred the right lower limb (78.1%). The players’ experience was also a factor to be considered, measured by the number of years they had played in a senior squad. The inclusion criteria for the analysis required players to be officially registered as male professional footballers from this single club for at least 6 months of the four analyzed seasons, with complete data for internal (RPE) and external (GPS-derived) workload variables. Players were excluded from the analysis for specific periods where data completeness was below 90%. Still, the study was conducted using a longitudinal time-window approach, meaning that the total sample comprises all available player-exposure time-windows over the four years, rather than a fixed cohort investigation. This approach mitigates the effect of player turnover, as each sampling window is treated as an independent event for model training and development. The total number of unique players contributing data was N = 96. All the applied procedures were approved by the Faculty of Human Kinetics Ethics Committee (CEIFMH No. 34/2021). The research was conducted in accordance with the principles outlined in the Declaration of Helsinki, and all participants provided informed consent forms before participating in this study.

### 2.2. Sports Data

This study was conducted in a single male professional football club competing in the Portuguese Professional Football Leagues during four consecutive sporting seasons (i.e., from 2020/2021 to 2023/2024). The description of the variables and the basic statistics for each variable incorporated in the prediction are presented in Table 1. The predictive models were calculated based on one dependent variable (y) and 14 independent variables (x1 through x14). The dependent variable is categorical, representing the occurrence of players’ injuries (0 = no injury; 1 = injury). The independent variables include players’ information (age, experience), external workload variables (WT, TD, Z4, Z5, Z6, HSR, ACC, DEC, PL, M/Min, MaxVel), and internal workload indicators (RPE). The predictive models were built by integrating data from 96 male professional football players, as they all performed the assessments while representing this professional football club.

This research was designed according to three methodological sets of assessments: (i) average season performance, (ii) two weeks’ performance before injury, and (iii) four weeks’ performance before injury. The average season performance dataset calculated the average of all external and internal loads per season and player. In the two weeks before the occurrence of all muscle injuries, external and internal loads per season and player were used. The last dataset comprised the same variables as the previous dataset, with the difference that this one included the performance of the four weeks preceding the occurrence of all muscle injuries across the four analyzed seasons. Data quality control was primarily maintained through device calibration and the implementation of a rigorous data filtering protocol. As detailed in the Participants section, time windows where data completeness fell below 90% were excluded from the analysis, mitigating the risk of including unreliable data influenced by signal loss from stadium structures or adverse environmental conditions. Still, players’ age and experience as professional players at the senior level were also introduced into all datasets. According to results of the Mann–Whitney non-parametric U test (Table 2), and subsequent adjustment for multiple testing using the Holm–Bonferroni correction calculation, no variables demonstrated statistically significant differences between the injured and non-injured groups (*p*_adj_ > 0.05 for all comparisons). However, by assessing the effect size (r), we identified differences of small practical relevance (r ≥ 0.20) for certain variables. Specifically, age and experience showed consistent minor effects across all three time windows (r = 0.20–0.22), suggesting that more experienced and older players may be at a slightly higher baseline risk. Load variables also demonstrated minor effects, particularly total distance (r = 0.23) and Z4 (high-speed running, 14–18 km/h) (r = 0.24) in the 4-weeks-before-injury window, and decelerations (r = 0.20) in the Season Average data. Since the group differences, as determined by basic statistics, were not statistically significant after adjustment, we shifted our focus to calculating predictive modeling using machine learning. This artificial intelligence (AI) approach might offer a more substantial and reliable investigation of the injury prediction phenomenon by modeling complex, non-linear interactions between variables with varying practical effect sizes.

### 2.3. Predictive Modeling

The predictive dataset was constructed based on a longitudinal time-window sampling approach. Each instance in the dataset represents a 2-week or 4-week period of player exposure, independent of whether the player completed the full four seasons. This methodology allowed us to maximize the available data instances and mitigate the effects of squad turnover. While data completeness was high (instances with <90% completeness were excluded during sampling), any remaining isolated missing data values were handled using the imputation feature of the Waikato Environment for Knowledge Analysis (WEKA) software version 3.9.6, replacing the missing values with the mean of that specific variable. The dependent variable (y) is categorical, representing the occurrence of a time-loss muscle injury. The positive class (“injury”) was defined by the workload data collected during the 2 or 4 weeks immediately preceding the occurrence of this specific event. In terms of the negative class (“non-injury”), it comprised all other player-exposure time-windows where no injury occurred in the subsequent period. If a player sustained multiple muscle injuries during the study period, each injury event was treated as an independent case. Data sampling for a subsequent event resumed only after the player was medically cleared to return to complete training/competition. All analyzed models were calculated in WEKA. WEKA is a software for learning and validating various predictive methods implemented in additional packages [36]. The study utilized predictive models to classify the occurrence of injuries. The models’ inputs represent individual input variables (x1–x14, Table 1), while the output represents the occurrence of an injury (y, Table 1). The classification methods used to calculate the models are reported in Table 3.

For the IBk classifier, the number of neighbors (k) was fixed at 1, in accordance with WEKA’s default configuration, as this setting provided stable and consistent performance during preliminary testing. The MLP classifier was implemented with a single hidden layer, in which the number of neurons was determined by the heuristic formula (number of attributes + number of classes)/2, following WEKA’s standard design. The learning rate and momentum were set to 0.3 and 0.2, respectively, and training was conducted for 500 epochs. No further hyperparameter tuning was performed, as the objective of this study was to evaluate and compare the baseline predictive performance of widely used classification algorithms under standardized conditions [36]. To mitigate class imbalance, the SMOTE filter (200% oversampling) was applied before model training, ensuring proportional representation of injury and non-injury cases across folds.

The models presented in the study were evaluated using cross-validation. This method assesses the quality of a model by dividing the data into two subsets: training and testing (or validation). There are many variations of cross-validation based on how the validation subsets are created. In the study, a 10-fold cross-validation method was employed. This method aims to divide the dataset into 10 training subsets. Each training set removes one-tenth of a 10-subset of the dataset, which becomes the testing set. Then, a model is constructed for nine other subsets, resulting in 10 versions of the model, which are evaluated by determining the error for the removed testing subset. The cross-validation errors (accuracy) for the models were calculated using the formula:

$$accuracy=\;\sum_{i=1}^{10}\frac{correct\;predictions}{all\;predictions}\cdot 100\;[\%]$$

where: i-step of cross-validation.

Additionally, auxiliary measures were calculated, i.e., balanced accuracy, sensitivity, specificity, precision, and F1-score, with the following formulas:

$$Balanced\;accuracy=\;\frac{sensitivity+specificity}{2}$$

$$Sensitivity=\frac{True\;Positives}{\left(True\;Positives+False\;Negatives\right)}$$

$$Specificity=\frac{True\;Negatives}{\left(True\;Negatives+False\;Positive\right)}$$

$$Precision=\frac{True\;Positives}{\left(True\;Positives+False\;Positives\right)}$$

$$F1-score=2\times \frac{\left(Precision\times Recall\right)}{\left(Precision+Recall\right)}$$

### 2.4. Injury Data

This study followed the recommendations of the Union of European Football Associations (UEFA) for epidemiological investigations [8]. An injury was defined as an event during a scheduled training session or match resulting in an absence from the next training session or match [8]. The medical department performed daily injury records during the seasons (training sessions and official matches), with all data initially stratified according to the standard UEFA criteria (e.g., location, type, severity). The club’s head physiotherapist consistently performed all injury diagnoses and maintained daily time-loss records, validating them using a standardized clinical assessment, strictly adhering to the UEFA definition of injury. For the predictive modeling, only time-loss muscle injuries were included in the final dataset, thus excluding traumatic injuries, concussions, and other time-loss illnesses. Injured players were monitored until they had completed their recovery period at the end of the season. The frequency, number of injured players, average number of days of injury per player, occurrence, exposure, and incidence were recorded and selected for analysis.

Regarding the variables under analysis, injury occurrence is characterized by the work session (training or competition) during which the athlete performed when the injury was sustained. The exposure time of the athletes throughout the seasons was collected using a 10 Hz GPS device during each training session and an official match. The injury incidence was calculated as the number of injuries sustained during a sporting activity divided by 1000 h of exposure time, multiplied by the time spent collecting data with the GPS device in-game and training situations.

### 2.5. Global Positioning System

The daily external load data of professional football players were monitored using two Global Positioning System (GPS) devices: a 1st–season 10-Hz GPS unit, EVO (Catapult, Melbourne, Australia), and a 10-Hz GPS unit, Apex pro series (STATSports, Newry, Northern Ireland, UK) used in the 2nd, 3rd, and 4th seasons. Data were collected over four competitive seasons, each spanning 46 weeks. To prevent device-specific effects from influencing the prediction outcomes of this study, we made the following methodological decisions: (i) the same intensity intervals were set to reduce possible differences in the GPS data collection; (ii) the analysis was designed to focus on intra-player comparisons; (iii) external load variables were normalized (Z-scores) for each player across all season; and (iv) data instances within the critical time windows for prediction (2 and 4 weeks before injury) were consistently collected using the same device type for each player. In terms of variables retained for analysis, we used the work time (WT) of the season, total distance (TD) covered, high-speed running (HSR), number of accelerations (ACC) and decelerations (DEC), and distance covered in high-speed zones (Z4, Z5, and Z6). It is important to note that the variables Z4, Z5, and Z6 represent the total accumulated distance (in meters) the player covers within the respective velocity zone. Table 4 presents the precise GPS metrics, units of measure, and velocity zones considered in this study. A skin-tight bag containing the GPS gadget was placed in the thoracic region between the scapulae. The GPS devices completed the placement, collection, and verification of the data that the club’s physical trainer recorded daily.

### 2.6. Rate of Perceived Exertion

The perceived exertion (RPE) registration rate occurred approximately fifteen minutes after each training session and official match ended. All players were assigned a numerical classification based on their perception of effort, where zero corresponded to no fatigue and 10 to extreme fatigue (i.e., Borg Scale CR10) [43,44]. All the records were made through the club’s mobile application or record sheets designed as a preventive tool at the locker room entrance.

## 3. Results

Regarding injury reports across the four sports seasons (Table 5), the included professional football players sustained a total of 97 injuries. The 2022/2023 season stands out for having suffered almost double the number of injuries (i.e., 39) than the other sports seasons analyzed. Even so, the number of injured players did not vary significantly between seasons, indicating that the injuries in the 2022/2023 season were mostly recurrence related. While the first two seasons had an average of approximately one injury per player, the 2022/2023 season once again stood out with an average of 1.5 injuries per player in the squad. Overall, most of the injuries occurred in training situations, with approximately the double of occurrences in competition moments (i.e., 64 vs. 33). However, the incidence of sports injuries was considerably four times higher in matches (IR: 12.3 per 1000 h; 95% CI: 8.57–17.30) than in training (IR: 3.0 per 1000 h; 95% CI: 2.33–3.88), since the exposure time in training sessions was substantially higher than in matches across the study timespan (21,340 h vs. 2679 h).

Table 6 summarizes the classification accuracy of all predictive models that forecast the occurrence of muscle injuries. Since injury prediction is inherently an imbalanced classification problem, the analysis focused on highlighting the AUC-PR, F1-score, and balanced accuracy metrics to ensure a robust evaluation. Across the three datasets and eight classifier algorithms, the Kstar classifier demonstrated the best performance, achieving an AUC-PR of 83% and a balanced accuracy of 72% when the model was trained on data from four weeks preceding each muscle injury. In terms of the predictive classifier with the best F1-score and balanced accuracy, it was the MLP classifier in the two-week window before each injury occurred, demonstrating an F1-score of 71% and a balanced accuracy of 72%. This model also verified a high ability to correctly identify injuries (sensitivity = 75%) and non-injuries (specificity = 69%).

## 4. Discussion

This longitudinal study aimed to analyze the differences between injury incidence during training sessions and matches and to build and classify different predictive models of injury risk based on players’ internal (RPE) and external (WT, TD, ACC, DEC, HSR, Z4, Z5, Z6) loads across four sports seasons. The classification method that showed the highest accuracy in predicting injury occurrence in this male professional football club was the Kstar classifier, achieving an AUC-PR of 83% and a balanced accuracy of 72% within the sample. This method was developed based on the internal and external loads of the variables above, four weeks before each muscle injury occurrence across the study period.

In our study, although the absolute number of injury occurrences was higher during training sessions (64 vs. 33), the injury incidence rate (per 1000 h of exposure) was nearly four times higher in matches (12.3) compared to training sessions (3.0). This finding is consistent with the literature, as identical conclusions have been observed in several investigations in professional football, where the likelihood of injury increases approximately five times during matches [13,45,46,47,48]. These results suggest that there should be a greater emphasis on prevention during the competitive period, including regular monitoring of players’ health and planning training weeks tailored to each player’s physical needs and limitations. Indeed, professional football players are frequently monitored and assessed by clubs’ clinical and coaching staff, aiming to prevent exposure-related conditions and consequently optimize players’ development, availability, and performance [49]. From a practical standpoint, these results reinforce the need to increase investment in preventive work during training, ensuring that training loads progressively replicate competitive demands. Additionally, weekly planning should be individualized, with regular monitoring and adjustments to balance recovery, physical preparation, and injury risk reduction throughout the competitive period.

In building the classification models, we assessed external load variables, including training and playing time, total distance, high-speed running, the number of accelerations and decelerations, and speed peaks, which players reach at significant km/h marks. Sports scientists have worked nonstop to evaluate the relationship between injuries and workload performance indicators [50,51,52]. Consequently, scientific evidence has concluded that the most relevant external load metrics to analyze in terms of professional players’ demands are those that demonstrate the ability to express the intensity, volume, and physical effort of the players (WT and TD) and the frequency and intensity of their speed changes (HSR, ACC, DEC, Z4, Z5, Z6) [53,54]. However, a longitudinal study conducted with the participation of 224 football players of different ages and competitive levels concluded that there are still unknowns regarding the relationship between external load variables and their impact on injury occurrence [55]. Even though sports science literature has verified that designing training programs with external loads is a crucial factor for injury prevention and players’ physical optimization [14,56,57], considering players’ perceptual response to the workloads, frequently measured by RPE, can increase the validity of the practical reflections drawn [58]. In other words, integrating RPE and GPS-derived load metrics provides a more comprehensive framework for monitoring training load, as it captures both the external mechanical demands and the individual physiological and perceptual responses to those demands. This combined, multivariate approach enables the models to interpret the complex, non-linear relationship between stress and strain more effectively [23,59]. Crucially, the validity of this integrated approach relies entirely on the standardization of data collection protocols (e.g., consistent RPE collection timing, standardized GPS settings) within the club, which was strictly maintained throughout the four-season study period [60]. The combination of internal and external loads can provide improved insights for coaching and clinical sport science staff to inform decisions regarding players’ physical abilities and those who may be more susceptible to muscle injury in a short period [61]. Furthermore, the literature suggests that the combined use of RPE and GPS is a viable and effective strategy for monitoring total load, allowing for individualized adjustments and a deeper understanding of the demands placed on athletes and their responses [23,59,60,61].

After building the classification methods based on the most significant variables of internal and external loads, one of the critical factors for predicting a sports injury is the timeline and workload leading up to its occurrence. A recent scientific investigation has reinforced the notion that the timeframe preceding an injury can significantly influence the load-injury relationship and prediction [55]. Many investigations have predicted this concern, but the literature lacks consistency regarding the most suitable temporal correlation between load and injury [50,52]. Our research verified that the Kstar classifier achieved the best overall predictive performance, with an AUC-PR of 83% and a balanced accuracy of 72% when using internal and external load data from the four-week window preceding each muscle injury. Nevertheless, within the two-week window, the MLP classifier showed an F1 Score of 71% and a balanced accuracy of 72%. Its high detection capability, achieving a sensitivity of 75% and a specificity of 69%, confirms its usefulness as an early warning decision support tool, maintaining an appropriate balance between correctly identifying possible risks of players sustaining muscle injuries and minimizing false alarms. Several investigations into the injury prediction of professional footballers using external and internal loads have been conducted over the past decade [17,62,63,64]. One of these recent investigations proposed a multidimensional injury forecasting approach using GPS data and machine learning techniques [62]. The authors verified that TD could detect around 80% of the injuries with about 50% precision [62]. TD’s high detection rate (Sensitivity) is likely because it is a primary indicator of Total Training Volume and Accumulated Mechanical Stress, meaning nearly all injury events were preceded by high TD exposure. However, this high detection rate was accompanied by low precision (50%), meaning half of the predictions were false positives, significantly limiting its practical utility in clinical decision-making. Quantitatively, our MLP model achieved superior precision (67%) while integrating both internal and external load features, demonstrating a more balanced predictive capability with a superior precision outcome compared to isolated TD.

Another study, conducted with 40 male professional football players, aimed to combine external and internal load features to predict injuries using classification algorithms that perform best on these features [17]. The results demonstrated that the internal load feature was more accurate than the external load one week before the injury occurred. Despite this, in forecasting sports injuries using a dataset with a one-month timespan, the classifiers showed better accuracy when internal and external loads were combined [17]. Thus, the literature continues to show significant differences, with different studies showing superior accuracy in predicting the occurrence of injuries in professional football using different relationship time loads. Reflecting in practice, the results obtained in injury prediction through machine learning techniques should not be generalized to samples other than the one used to build them. Instead, they should be viewed as fundamental insights for attention and prevention, highlighting potential injury predictors that can soon impact the availability of professional footballers. Assuming that different samples of professional football players have distinct physical characteristics and require tailored work methodologies, technical and clinical staff should be adapted accordingly. Technical teams should consider the predictors and models previously built as insights for attention and prevention for their target population.

Recent advancements in sports injury prediction have increasingly focused on hybrid AI models and deep learning techniques. These are well recognized for capturing the complex, sequential, and temporal patterns of the training load data [65,66,67]. Studies show that Long Short-Term Memory networks and other deep learning models outperform traditional machine learning algorithms (such as RBF and Random Tree) in predictive accuracy for injury risk, with accuracy rates frequently above 90% [65,66]. Hybrid models combining Convolutional Neural and Long Short-Term Memory networks further enhance the ability to extract spatial and temporal features from athlete data, leading to more robust injury prediction [67]. Explainable AI has also become a critical area of focus as these methods are increasingly used to provide transparency, allowing clinical staff to understand the rationale behind AI models’ insights. Indeed, the literature emphasizes that integrating Explainable AI is crucial for achieving clinical utility, ensuring regulatory compliance, and facilitating effective interventions in sports science [68,69].

The findings of this study must be interpreted in light of certain limitations. The omission of information regarding players’ past injury histories, cumulative load measures, and contextual variables such as player position or minutes played per player represents a limitation, given the widely recognized influence of these variables on a player’s propensity to sustain new muscle injuries during a sports season. For instance, omitting players’ previous injury history is a significant limitation, as the literature consistently identifies prior injury as the strongest single predictor of a future muscle injury in football and other sports. Systematic reviews and extensive cohort studies demonstrate that athletes with a history of muscle injury have a significantly higher risk of sustaining a subsequent injury, with hazard ratios and odds ratios often exceeding 2.0 for recurrence or a similar injury type [70,71]. For example, players injured in one season are more than twice as likely to be injured in the following season as those without a prior injury [71]. By excluding this critical, high-impact variable, our models are limited to predicting risk based only on recent workload exposure (acute load data), without incorporating individual susceptibility or the cumulative biological risk from previous tissue damage. Secondly, the model training and validation data were sourced exclusively from a single professional football club. This lack of external validation means the models are inherently limited in generalizability to other teams or leagues operating under different training and competitive environments. Finally, our models were validated using cross-validation (within-sample) rather than prospective validation (real-time prediction on future, unseen data). While cross-validation is robust, it cannot fully confirm the model’s clinical utility or predictive performance in a real-world scenario and can lead to overfitting. Future research should prioritize the inclusion of comprehensive injury history, alongside external and prospective validation, to establish the clinical relevance of these predictive models.

Nevertheless, achieving robust predictive performance using 4-year longitudinal data from a professional football context, encompassing both internal (RPE) and external (GPS-derived) load metrics, strengthens the link between theoretical evidence and practical application. Notably, the four-week time span was the most accurate window for the overall best-performing model (Kstar classifier), with an AUC-PR of 83% and a balanced accuracy of 72%. This finding underscores the importance of assessing accumulated chronic load in identifying underlying risk, a key requirement for proactive load management. Even so, the two-week time span also demonstrated high predictive capability, particularly with the MLP classifier, which showed an F1-score of 71% and a balanced accuracy of 72%. These results confirmed that a shorter, more acute analysis window remains highly relevant for predicting immediate and near-term injury risk in this specific cohort of professional football players. The overall high performance, particularly in terms of AUC-PR, confirms the utility of these workload metrics as early warning decision support tools, despite the mentioned limitations.

This investigation thus supports the notion that predictive models combining internal and external workloads can significantly aid coaching and clinical staff members in making more informed decisions about players’ physical capabilities and availability to train and compete. In practical terms, this methodology provides initial data that may support, but not replace, clinical and coaching decisions, offering insights to tailor interventions and guidance to particular players who are more susceptible to muscle injuries at a given point in the season. Future sports science research could benefit from an investigation into the qualitative vision of coaches and technical teams regarding the in-field applicability of predictive models.

## 5. Conclusions

This investigation demonstrated that predictive models combining internal (RPE) and external (GPS-derived) workloads achieved robust predictive performance over the two- and four-week prediction windows for muscle injuries in this cohort of professional football players. Specifically, the Kstar classifier (4-week window) provided the strongest results, with an AUC-PR of 83% and a balanced accuracy of 72%. These findings suggest a significant potential for developing valuable, data-driven early-warning systems based on integrated load data, particularly by leveraging models capable of balancing high sensitivity (correct injury identification) and specificity (correct non-injury identification).

## Figures and Tables

**Table 1:** Description of the variables used to construct the predictive models (N = 14).

Variable	Description	Average Season		2 Weeks Before Injury		4 Weeks Before Injury	
		M ± SD		M ± SD		M ± SD	
x1	Age (y)	26.22 ± 4.19		26.22 ± 4.19		26.22 ± 4.19	
x2	Experience (y)	8.16 ± 4.18		8.16 ± 4.18		8.16 ± 4.18	
x3	WT (min)	73.17 ± 6.14		74.55 ± 9.18		74.71 ± 8.32	
x4	TD (m)	4933.09 ± 559.41		5222.95 ± 1083.50		5223.06 ± 1030.41	
x5	Z4 (m)	528.60 ± 94.70		569.69 ± 170.32		565.50 ± 149.36	
x6	Z5 (m)	311.68 ± 68.69		324.11 ± 93.35		321.31 ± 85.85	
x7	Z6 (m)	57.54 ± 21.52		57.10 ± 26.25		57.22 ± 24.63	
x8	HSR (m)	367.89 ± 84.78		380.39 ± 110.47		374.34 ± 99.16	
x9	ACC (m)	42.03 ± 12.92		43.57 ± 14.60		42.96 ± 13.43	
x10	DEC (m)	42.46 ± 11.72		43.72 ± 14.76		42.79 ± 13.06	
x11	PL (m)	221.41 ± 173.41		222.48 ± 179.26		224.00 ± 181.42	
x12	M/Min (m)	75.32 ± 9.01		76.06 ± 12.33		75.86 ± 11.83	
x13	MaxVel (m)	27.07 ± 1.35		27.20 ± 1.72		27.35 ± 2.07	
x14	RPE	4.91 ± 0.86		4.99 ± 0.96		4.97 ± 0.90	
y	Injury Occurrence *	NI (69)	I (27)	NI (69)	I (59)	NI (69)	I (59)

* categorical variable (NI = non injury; I = injury), M (mean value), SD (standard deviation), y (year), min (minutes), m (meters), WT (work time), TD (total distance), Z4 (14–18 km/h), Z5 (18–24 km/h), Z6 (24–39 km/h), HSR (high-speed running), ACC (accelerations), DEC (decelerations), PL (player load), M/Min (meters per minute), MaxVel (maximal velocity), RPE (rate of perceived exertion). Z4, Z5, and Z6 represent the total accumulated distance in meters the player covers within the respective velocity zone.

**Table 2:** Descriptive statistics according to three methodological sets of assessments to differentiate injured and non-injured player profiles in external and internal loads.

Variable	Season Average							4 Weeks Before Injury Occurrence						2 Weeks Before Injury Occurrence					
	Group	M	SD	Mdif	*p*_adj_	r	Rmag	M	SD	Mdif	*p*_adj_	r	rmag	M	SD	Mdif	*p*_adj_	r	rmag
Age (y)	NI	25.67	3.79	1.96	>0.05	0.20	Small	25.67	3.79	1.96	>0.05	0.20	Small	25.67	3.79	1.96	>0.05	0.20	Small
	I	27.63	4.87					27.63	4.87					27.63	4.87				
Experience (y)	NI	7.57	3.76	2.10	>0.05	0.22	Small	7.57	3.76	2.10	>0.05	0.22	Small	7.57	3.76	2.10	>0.05	0.22	Small
	I	9.67	4.85					9.67	4.85					9.67	4.85				
WT (min)	NI	73.50	5.82	1.19	>0.05	0.16	Small	73.50	5.82	2.62	>0.05	0.10	Small	73.50	5.82	2.27	>0.05	0.10	Small
	I	72.31	6.91					76.16	10.40					75.77	11.91				
TD (m)	NI	4981.17	549.08	170.98	>0.05	0.15	Small	4981.17	549.08	524.77	>0.05	0.23	Small	4981.17	549.08	524.53	>0.05	0.18	Small
	I	4810.19	577.16					5505.94	1349.22					5505.70	1437.45				
Z4 (m)	NI	530.93	89.19	8.28	>0.05	0.04	Negligible	530.93	89.19	75.00	>0.05	0.24	Small	530.93	89.19	84.08	>0.05	0.23	Small
	I	522.64	109.14					605.93	190.89					615.01	224.31				
Z5 (m)	NI	315.64	71.45	14.07	>0.05	0.05	Negligible	315.64	71.45	12.30	>0.05	0.09	Negligible	315.64	71.45	18.37	>0.05	0.10	Small
	I	301.57	61.15					327.94	100.35					334.01	113.63				
Z6 (m)	NI	58.79	22.27	4.43	>0.05	0.09	Negligible	58.79	22.27	3.41	>0.05	0.09	Negligible	58.79	22.27	3.66	>0.05	0.11	Small
	I	54.36	19.50					55.38	27.21					55.13	30.33				
HSR (m)	NI	373.90	88.17	21.38	>0.05	0.09	Negligible	373.90	88.17	0.95	>0.05	0.01	Negligible	373.90	88.17	14.08	>0.05	0.07	Negligible
	I	352.52	74.92					374.85	111.48					387.98	132.33				
ACC (m/s^2^)	NI	42.94	13.14	3.21	>0.05	0.08	Negligible	42.94	13.14	0.05	>0.05	0.01	Negligible	42.94	13.14	1.37	>0.05	0.05	Negligible
	I	39.73	12.25					42.99	13.87					44.31	16.23				
DEC (m/s^2^)	NI	43.93	11.45	5.23	>0.05	0.20	Small	43.93	11.45	2.47	>0.05	0.11	Small	43.93	11.45	0.46	>0.05	0.06	Negligible
	I	38.70	11.78					41.46	14.71					43.47	17.98				
PL (AU)	NI	224.33	172.26	10.38	>0.05	0.07	Negligible	224.33	172.26	0.72	>0.05	0.05	Negligible	224.33	172.26	4.02	>0.05	0.05	Negligible
	I	213.95	179.41					223.61	193.07					220.31	188.58				
M/min (m/min)	NI	75.24	8.75	0.28	>0.05	0.03	Negligible	75.24	8.75	1.35	>0.05	0.10	Small	75.24	8.75	1.78	>0.05	0.08	Negligible
	I	75.52	9.82					76.59	14.69					77.02	15.53				
MaxVel (m/s)	NI	27.17	1.36	0.35	>0.05	0.12	Small	27.17	1.36	0.39	>0.05	0.06	Negligible	27.17	1.36	0.05	>0.05	0.08	Negligible
	I	26.82	1.31					27.56	2.68					27.22	2.08				
RPE	NI	5.01	0.87	0.35	>0.05	0.19	Small	5.01	0.87	0.09	>0.05	0.05	Negligible	5.01	0.87	0.03	>0.05	0.02	Negligible
	I	4.66	0.79					4.92	0.94					4.98	1.06				

I—injury group; NI—non-injury group; M—mean; SD—standard deviation; *p*_adj_—*p*-value adjusted by the Holm–Bonferroni correction; r—effect size: Interpreted according to Cohen’s criteria [35]: r ≥ 0.5 (Large), r ≥ 0.3 (Medium), r ≥ 0.1 (Small). r < 0.1 (Negligible); rmag—magnitude of effect size; y—years; min—minutes; m—meters; m/s—meters per second; AU—arbitrary unit; m/min—meters per minute; WT—work time; TD—total distance; Z4—14–18 km/h; Z5—18–24 km/h; Z6—24–39 km/h; HSR—high-speed running; ACC—accelerations; DEC—decelerations; PL—player load; MaxVel—maximal velocity; RPE—rate of per-ceived exertion.

**Table 3:** Description of the classification methods used to calculate the models.

IBk	IIBk is an implementation of the KNN classifier that uses a distance function. By default, k = 1, meaning it considers only one nearest neighbor. The number of neighbors can be set manually (-K) or automatically through leave-one-out cross-validation (-X). If more neighbors are selected, their predictions can be weighted based on their distance to the test instance. There are two different formulas for calculating the weight from the distance (-D and -F). The time required to classify a test instance with the nearest neighbor classifier increases linearly with the number of training instances [37].
K-Star	IK-Star is a type of instance-based classifier where the class of a test instance is determined by the classes of similar training instances, identified through a similarity function. Unlike other instance-based classifiers, K-Star employs an entropy-based distance function [38].
Simple Logistic	Simple Logistic is a classification algorithm used for predictive modeling when the target variable is categorical. It is particularly useful for binary classification problems, where the output is either one class or another (0 or 1). A threshold is set to determine the class assignment based on the predicted value [39].
Logistic Classifier	Logistic Classifier is a modified logistic regression method. Unlike the classical method, in this model I realize the handling of instance weights. The class implementing this model uses a ridge estimator [40].
MLP Classifier	MLP Classifier is an artificial neural network with one hidden layer. The process of calculating and optimization involves minimizing a loss function with a quadratic penalty using the BFGS method, with all attributes standardized. Key parameters include the ridge parameter for weight penalty and the number of hidden units, which affects training time. The default activation function for the hidden layer is an approximate logistic function, but the output layer uses the sigmoid function for classification. Nominal attributes are converted to binary, and missing values are globally replaced [36].
Random Tree	Random Tree is an advanced technique for building tree-based classifiers. Used in supervised learning for both classification and regression tasks. By employing multiple classification or regression trees and incorporating randomness, this method generates predictions that are highly resilient to new data [41].
RBF Classifier	Radial basis function networks are a type of feedforward network. An RBF (Radial Basis Function) network is characterized by the hidden neuron implementing a function, called the basis function, which varies radially around a selected center [41].
SMO	Sequential Minimal Optimization is a simple algorithm that can quickly solve the Support Vector Machines quadratic programming (QP) problem without any extra matrix storage. SMO decomposes the overall QP problem into QP sub-problems, using Osuna’s theorem to ensure convergence [42].

IBk (Instance-Based K), MLP (Multi-Layer Perceptron), RBF (Radial Basis Function), SMO (Sequential Minimal Optimization).

**Table 4:** GPS metrics measured for four sports seasons, their units, and zones.

Metric	Unit	Zone
Work time	Minutes	
Total distance	Meters	
Accelerations		Above 10.8 km/h
Decelerations		Above 10.8 km/h
High-speed running		Above 18 km/h
High-speed zone 4		14–18 km/h
High-speed zone 5		18–24 km/h
High-speed zone 6		24–39 km/h

**Table 5:** Injury characterization during four sportive seasons.

Sportive Season	2020/2021	2021/2022	2022/2023	2023/2024	Total
Club (No.)	21	23	26	26	96
Injured Players	12	13	14	10	49
Injury Frequency	21	22	39	15	97
Injuries per player (Av.)	1	0.9	1.5	0.6	1
Injury Occurrence (No.)					
Training	13	15	25	11	64
Match	9	6	14	4	33
Injury Exposure (h)					
Training	4011	5245	5204	6880	21340
Match	440	619	970	650	2679
Injury Incidence (per 1000 h)					
Training	4.0 (1.72–5.54)	2.9 (1.60–4.72)	4.8 (3.11–7.18)	1.6 (0.80–2.86)	3.0 (2.33–3.88)
Match	20.5 (9.35–38.83)	9.7 (3.55–21.71)	14.4 (7.89–24.22)	6.2 (1.67–15.75)	12.3 (8.57–17.30)

No. (number), Av. (average), h (hours). The values in parentheses for Injury Incidence represent the 95% Confidence Interval (95% CI) calculated using the Poisson distribution method.

**Table 6:** Classification accuracy of predictive models for average season performance, two weeks before injury and four weeks before injury occurrence based on GPS and RPE.

Method	Dataset	Accuracy	Balanced Accuracy	Sensitivity	Specificity	Precision	F1-Score	AUC-ROC	AUC-PR
Logistic	2 Weeks Before Injury	55%	57%	76%	38%	51%	61%	67%	73%
	4 Weeks Before Injury	61%	62%	77%	46%	55%	65%	71%	75%
	Average Season	62%	62%	61%	62%	39%	47%	63%	56%
MLP	2 Weeks Before Injury	72%	72%	75%	69%	67%	71%	79%	80%
	4 Weeks Before Injury	68%	68%	73%	63%	63%	68%	75%	78%
	Average Season	60%	57%	51%	63%	35%	42%	59%	53%
RBF	2 Weeks Before Injury	64%	66%	88%	43%	57%	69%	77%	78%
	4 Weeks Before Injury	56%	58%	84%	33%	51%	64%	68%	72%
	Average Season	58%	56%	51%	61%	34%	41%	59%	53%
SMO	2 Weeks Before Injury	46%	49%	98%	1%	46%	62%	49%	46%
	4 Weeks Before Injury	46%	49%	96%	2%	46%	62%	49%	46%
	Average Season	62%	64%	67%	60%	40%	50%	63%	40%
Simple logistic	2 Weeks Before Injury	53%	55%	81%	29%	49%	61%	65%	70%
	4 Weeks Before Injury	59%	60%	80%	40%	53%	64%	71%	76%
	Average Season	61%	62%	63%	60%	38%	48%	64%	57%
IBk	2 Weeks Before Injury	70%	70%	69%	71%	67%	68%	70%	64%
	4 Weeks Before Injury	67%	67%	66%	68%	64%	65%	67%	62%
	Average Season	46%	42%	33%	51%	21%	25%	42%	29%
KStar	2 Weeks Before Injury	70%	70%	67%	73%	68%	67%	79%	81%
	4 Weeks Before Injury	73%	72%	69%	76%	71%	70%	81%	83%
	Average Season	52%	50%	46%	54%	28%	35%	48%	39%
Random Tree	2 Weeks Before Injury	67%	68%	74%	61%	62%	68%	67%	60%
	4 Weeks Before Injury	68%	69%	77%	61%	63%	69%	69%	61%
	Average Season	53%	46%	33%	60%	24%	28%	46%	31%

Accuracy (total proportion of correct predictions (both correctly identified injuries and correctly identified non-injuries) that the model made relative to the total number of predictions), balanced accuracy (arithmetic mean of sensitivity and specificity, used when dealing with imbalanced data), sensitivity (proportion of injured players who were correctly identified as injured by the model), specificity (proportion of non-injured players who were correctly identified as non-injured by the model), precision (quantification of the number of correct positive predictions made out of all the positive predictions made by the model), F1-score (statistical measure of the accuracy of an individual test), AUC-ROC (summarization of the trade-off between the true-positive rates (sensitivity) and the false-positive rates for a predictive model), AUC-PR (overall performance of binary classification models, mostly used when the classes are imbalanced).

## Data Availability

The data presented in this study are available upon request from the corresponding author.
